# *Lumicella*, a new genus of the tribe Empoascini (Hemiptera, Cicadellidae, Typhlocybinae) from China

**DOI:** 10.3897/zookeys.364.6618

**Published:** 2013-12-17

**Authors:** Si-han Lu, Li Zhang, Li Qiao, Dao-zheng Qin

**Affiliations:** 1 Key Laboratory of Plant Protection Resources and Pest Management of the Ministry of Education; Entomological Museum, Northwest A&F University, Yangling, Shaanxi 712100, China

**Keywords:** Homoptera, Auchenorrhyncha, leafhoppers, taxonomy, distribution

## Abstract

*Lumicella rotundata* **gen.** *et* **sp. n.** is described based on specimens from Fujian Province, China. Habitus photos and illustrations of male genitalia of this new species are provided. Differences between the new genus and closely related genera are discussed.

## Introduction

The fauna of Empoascini in China is very rich and diverse, this is associated with China’s high biodiversity. To date, 31 genera of this tribe have been described in Chinese fauna (Matsumura 1931; Dworakowska 1971, 1973, 1982, 1993, 1995; Zhang 1990; Qin 2003; Zhang and Qin 2004, 2005; Qin and Zhang 2003, 2008; Qin et al. 2010, 2011a, 2011b, 2013); Qin and Zhang (2008) provided a key to the genera of the tribe from China. However, our knowledge of the Chinese fauna of this tribe is still incomplete with many genera and species remaining to be described. In this paper, a new genus and species is described based on our recent examination of unidentified materials collected from southern China, as well as habitus photos and drawings of male genitalia of the new species.

## Material and methods

The specimens examined in this study are deposited in the Entomological Museum, Northwest A&F University, Yangling, Shaanxi, China (NWAFU). The entire male abdomen of the examined specimens were removed and cleared in 10% NaOH and drawn from preparations preserved in glycerin. External morphology was observed under an Olympus SZX-10 microscope. Photographs of the specimens were made using a Nikon SMZ 1500 microscope with a Retiga 2000R camera (CCD). Images were produced using the software Auto-Montage Pro. The male genitalia were drawn using a Olympus PM-10AD, and wings were drawn with a Leica MZ-12.5 microscope. All the pictures were edited and enhanced using Adobe Photoshop CS7.0 (Adobe Systems). The body measurements are from apex of the vertex to the tip of the forewing.

Morphological terminology predominantly follows Zhang (1990) except for the nomenclature of the wing and setae on the subgenital plate, where we follow Dworakowska (1993) and Southern (1982) respectively.

## Taxonomy

### Lumicella

Lu & Qin gen. n.

http://zoobank.org/1CF76EB3-99CB-441A-B1F4-10178954ED2A

http://species-id.net/wiki/Lumicella

#### Type species.

*Lumicella rotundata* Lu & Qin, sp. n., here designated.

#### Description.

**Figures 1–8.**
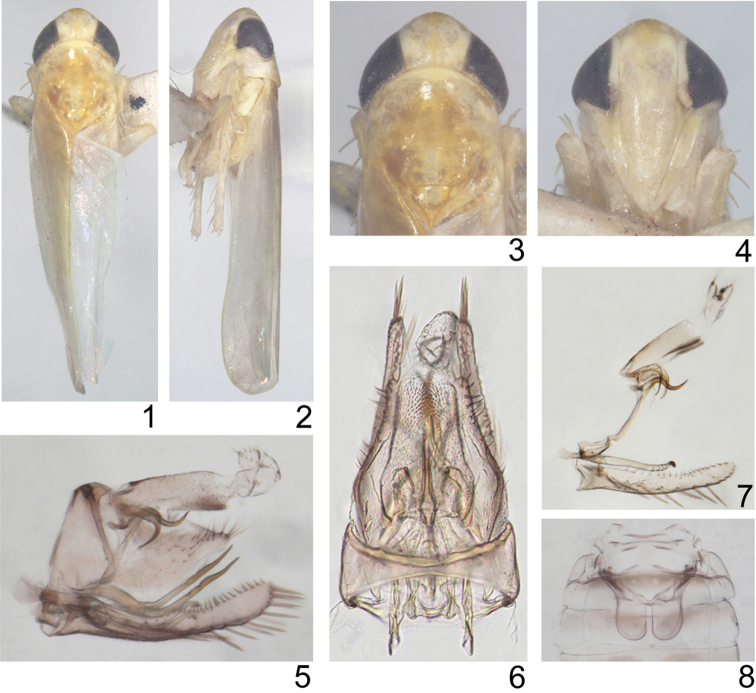
*Lumicella rotundata* sp. n. **1** male adult (abdomen removed), dorsal view **2** same, left lateral view **3** head and thorax, dorsal view **4** face **5** male genitalia, left lateral view **6** same, dorsal view **7** anal tube and anal styli, aedeagus, connective, paramere and subgenital plate, left lateral view **8** abdominal apodemes.

**Figures 9–20.**
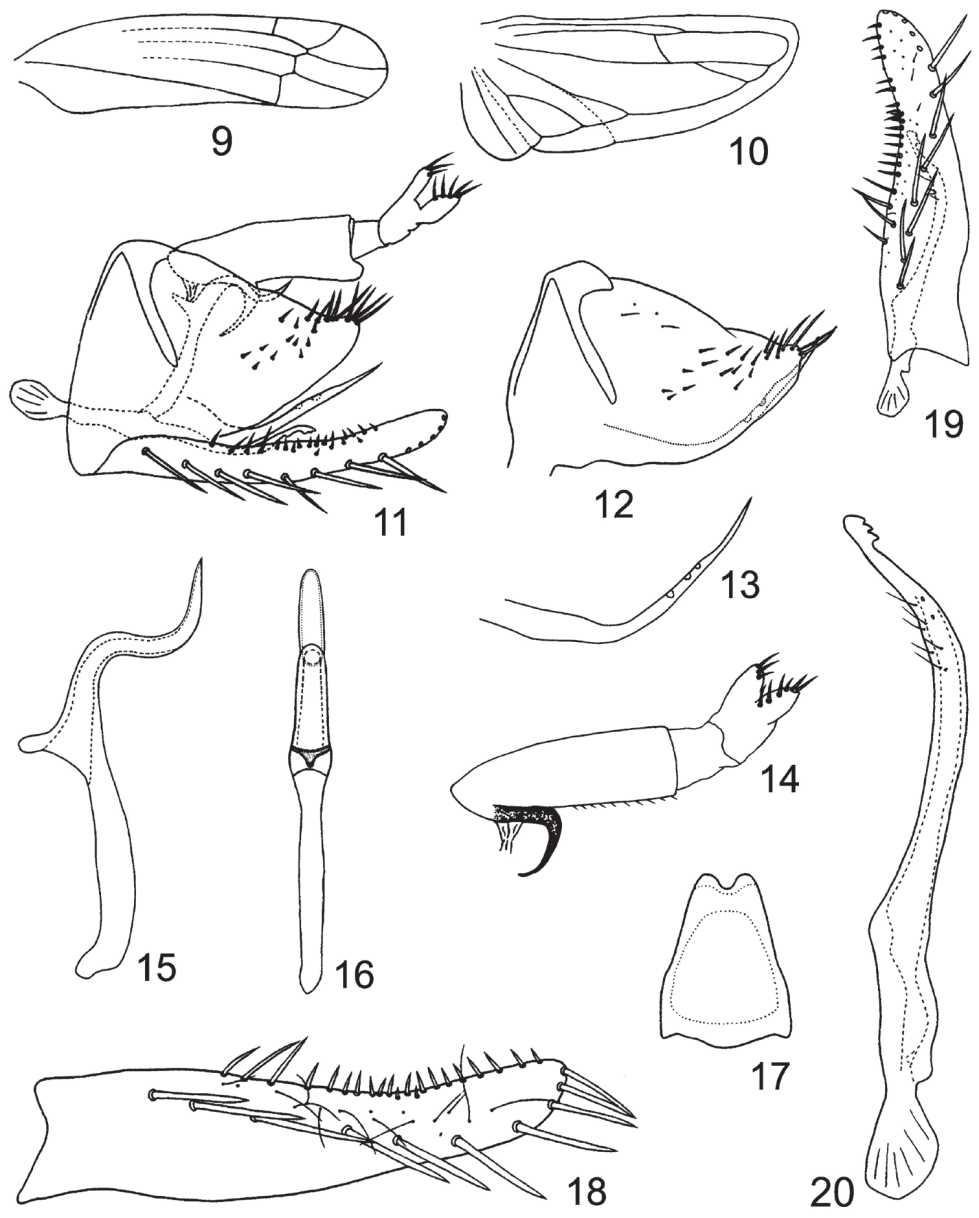
*Lumicella rotundata* sp. n. **9** forewing **10** hindwing **11** male genitalia, left lateral view **12**, pygofer side and ventral pygofer appendage, left lateral view **13** ventral pygofer appendage, left lateral view **14** anal tube and anal styli, left lateral view **15** aedeagus, left lateral view **16** same, dorsal view **17** connective **18** subgenital plate **19** subgenital plate and paramere, dorsal view **20** paramere.

Body small. Head with eyes broader than maximum width of pronotum (Figs 1, 3). Vertex short, rounded anteriorly (Figs 1, 3), profile of transition to face rounded (Fig. 2), coronal suture long (Figs 1, 3). Face narrow and slightly convex in profile, lateral frontal suture present (Figs 2, 4). Forewing narrow, rounded apically, apical cells occupying less than one-third total length, all apical cell with separate bases, 2nd apical cell with margins subparallel but slightly broadened at apex, c and r cells nearly equal in width, narrower than m and cua cells; veins RP, MP’ arise from r cell and MP”+CuA’ from m cell (Fig. 9). Hindwing with bifurcation point of CuA basad of coalescence of CuA with MP” (Fig. 10).

Male basal abdominal sternal apodemes developed, apically rounded and parallel sided (Fig. 8). Male pygofer elongate, strongly narrowing caudad, terminally with rigid microsetae on each side of lobe, ventral appendage present (Figs 5, 6, 11–13), dorsal bridge short, less sclerotized in middle dorsocaudad (Fig. 6). Subgenital plate much exceeding pygofer side, A-group setae distinct, C-group setae arranged in a single row and reaching apex of plate (Figs 5, 11, 18, 19). Paramere slim, apophysis bearing prominent dentifer and a few slender setae (Figs 5, 7, 11, 19, 20). Connective lamellate (Fig. 17). Aedeagus without dorsal apodeme, preatrium well developed, shaft tubular and curved twice, gonopore apical on ventral side (Figs 15, 16). Anal tube process curved and narrowed terminally (Figs 5, 7, 11, 14).

#### Etymology.

The generic name is an arbitrary combination of letters, and is regarded as feminine.

#### Discussion.

In *Alebroides* Matsumura group, the new genus is similar to *Ghauriana* Thapa, *Membranacea* Qin & Zhang, *Dattasca* Dworakowska, *Luvila* Dworakowska, *Szara* Dworakowska, *Szuletaia* Dworakowska, *Luodianasca* Qin & Zhang, *Nikkotettix* Matsumura and *Znana* Dworakowska in having veins RP, MP’ of forewing arise from r cell and MP”+CuA’ from m cell, all apical cells in fore wing having separate bases (in *Nikkotettix* and *Znana*, 3rd apical cell stalked or sessile) and CuA in the hindwing branched apically. However, this new genus differs from *Membranacea*, *Luodianasca*, *Luvila* and *Szara* in the presence of the ventral pygofer appendage (ventral pygofer appendage absent in these four genera), from *Dattasca* and *Szuletaia* in having bifurcation point of CuA basad of coalescence of CuA with MP” (apicad of coalescence of CuA with MP” in *Dattasca* and *Szuletaia*),from *Znana* in having coronal suture not reaching apex of vertex (surpassing apex of vertex and reaching the level of ocelli on face in *Znana*); from *Ghauriana* in the subgenital plate having A-group setae (A-group setae undifferentiated in *Ghauriana*), from *Nikkotettix* in the absence of ventral process at the base of aedeagal shaft (with ventral process at the base of aedeagal shaft in *Nikkotettix*). The new genus also differs from *Membranacea* in the presence of anal tube appendage (anal tube appendage absent in *Membranacea*) and from *Luvila* in having the C-group setae of subgenital plate arranged in a single row subbasally (C-group setae arranged in two rows subbasally in *Luvila*).

#### Distribution.

China (Fujian).

### Lumicella rotundata

Lu & Qin sp. n.

http://zoobank.org/771BE1DE-E369-4879-A4E0-08276BF30F46

http://species-id.net/wiki/Lumicella_rotundata

Figs 1 –20

#### Description.

Body length: Male 3.7–3.9mm.

General colour variable: lighter coloured specimens yellow to ochre-yellow. Vertex with borders at eyes creamy-yellowish, semilunar patch mesocaudad of ocelli creamy. Face and basal antennal segments light yellow. Eyes blackish-brown. Disc of pronotum golden-yellow, irregular arch of hypodermal pattern light-yellow in addition to three large creamy patches along anterior margin. Centre of scutellum sordid cream, scutoscutellar sulcus beige. Darker specimens brown to sordid brown, semilunar patch mesocaudad of ocelli, borders at eyes, genae, patches on pronotum and centrally on scutellum, sordid cream.

Male genitalia: Basal sternal abdominal apodemes exceeding half of segment 4 (Fig. 8). Male pygofer with about 16 rigid setae on outer and inner surface of lobe, ventral pygofer appendage slim and bent caudodorsad near base, surpassing caudal margin of lobe, tapering and sculptured with depressions subapically (Figs 5, 11–13). Subgenital plate with nearly same width in basal third, apical 2/3 gradually narrowing towards apex, A-group setae (3–4) rigid, B-group seate (15-17) small, roughly uniseriate along dorsal margin in apical half, C-group setae (13–14) arising near base of plate, sharply terminated, D-group setae roughly bi- or tri-seriate, starting caudad of C-group setae (Figs 5, 11, 18, 19). Paramere sinuate in caudal part, apically bearing 3 big teeth preceded by ca. 6 fine setae and few sensory pits (Figs 5, 11, 19, 20). Connective narrowing to deeply emarginate apex (Fig. 17). Aedeagal shaft tubular, longer than preatrium, in profile its middle part right-angled and curved caudoventrad followed by vertical apical region, gonopore large on ventral side, in ventral view aedeagus with rounded apex (Figs 5, 11, 15, 16). Anal tube process well sclerotized, originating subapically from ventral margin of anal tube, nearly reaching 1/3 height of pygofer (Figs 5, 11, 14).

#### Type material.

**Holotype.** ♂ (NWAFU), China, Fujian Province, Wuyi Mountain, 17 Aug 2008, coll. X. Gao and X. T. Li. **Paratypes.** 4♂♂(NWAFU), same data as holotype; 1♂(NWAFU), China, Fujian Province, Wuyi Mountain, 17 Sept 1980, coll. T. Chen; 10♂♂ (NWAFU), China, Fujian Province, Wuyi Mountain, 17 Aug 1984, coll. Z. X. Cui.

#### Etymology.

The name is derived from the Latin word “rotundus” (round), which refers to the rounded apex of the aedeagal shaft.

#### Distribution.

Known only from the type locality in Fujian Province in southeastern China.

#### Host plant.

Unknown.

## Supplementary Material

XML Treatment for Lumicella

XML Treatment for Lumicella rotundata
